# Analysis of Volatile Compounds with Odor Characteristics in Dianhong, Chuanhong, and Keemunhong Based on SPME-GC×GC-MS

**DOI:** 10.3390/molecules30102233

**Published:** 2025-05-21

**Authors:** Sinuo Li, Qi Meng, Chunli Huang, Peihan Zhou, Sirui Yao, Yamin Guo, Xiaojun Wang

**Affiliations:** 1 College of Food Science and Technology, Yunnan Agricultural University, Kunming 650201, China; 2 Yunnan Coffee Modern Industry College, Yunnan Agricultural University, Kunming 650201, China; 3 Laboratory of Molecular Sensory Science, School of Food and Health, Beijing Technology and Business University, Beijing 100048, China; mengqi@btbu.edu.cn (Q.M.);

**Keywords:** black tea, sensory evaluation, GC×GC-MS, OAV, key odor compounds

## Abstract

China is the place of origin and main producer of black tea worldwide, with Dianhong (DH), Chuanhong (CH), and Keemunhong (KH) being the famous Chinese black teas. The contents of various odor components in black teas differ with their origins. However, the effects of these differences on the presentation of distinctive odor characteristics in various products remain unclear. We aimed to elucidate the odor characteristics and odor compounds of these three black teas; to this end, we performed a sensory evaluation and multivariate statistical analysis based on comprehensive two-dimensional gas chromatography–mass spectrometry (GC×GC-MS) results. The sensory evaluation revealed that the odor characteristics of DH were floral and fruity, whereas sweet and herbal-like odors were more intense in CH and QH. A total of 119 volatile compounds were detected, with alcohols, aldehydes, and esters being the main volatile compounds. Among them, 41 volatile compounds were identified with an odor activity value (OAV) of >1, and 24 of them were selected through principal component analysis, hierarchical cluster analysis, and orthogonal partial least squares discriminant analysis as marker substances to distinguish the three teas; thus, 24 volatile compounds are important odor compounds of DH, CH, and QH.

## 1. Introduction

Black tea originates from Fujian Province, China, and the earliest black tea was called “Lapsang Souchong,” which has a history of more than 400 years and belongs to the category of fully fermented tea. Black tea was introduced in Europe in 1610 and has gradually become popular worldwide [1]. Several types of black tea are produced in China, including Dianhong (DH; originating from Yunnan), Keemunhong (KH; originating from Anhui), and Chuanhong (CH; originating from Sichuan). DH is made from Yunnan’s large-leaf varieties, and its unique and rich odor characteristics make it a representative Chinese black tea. KH comes from Anhui Province, and the raw material is selected from a mid-leaf and mid-season sprouting variety tea tree called “Chinkapin Leaf Species” (also known as Keemun species) with rich nutritional content. It is one of the top 10 famous teas in China, with a reddish and bright tea soup color, fresh and mellow taste, and rich and long-lasting aroma. CH is made from medium- and small-leaf tea leaves unique to local high-altitude mountain tea plantations in Yibin City, Sichuan Province. It exhibits exceptional quality characteristics, characterized by well-shaped leaves with distinct pekoe tips, presenting a lustrous black appearance. The intrinsic properties manifest as a highly aromatic profile and robust flavor intensity. Black tea is rich in carotene, vitamin A, polyphenols, trace elements, caffeine, various amino acids, and other nutrients that are beneficial for human health [2,3,4]. Further processed products of black tea include black tea paste, black tea jenny, and black tea food [5]. Unique compounds in black tea can extend the shelf life of the food, which has great potential for development in the food and healthcare industries.

Odor is one of the most intuitive senses that consumers associate with food [6]. Volatile compounds from fresh leaves and substances produced during processing lead to the odor composition of black tea [7], which is a key factor in deciding the quality and price of tea [8]. More than 400 volatile compounds have been detected in black tea, mainly alcohols, aldehydes, acids, ketones, esters, and heterocyclic compounds [9]. During black tea production, sugar glycoside hydrolysis, amino acid Strecker degradation, fatty acid catabolism, carotenoid oxidation, and the Maillard reaction are the main pathways that generate odor [10,11,12]. Some studies have shown that the liquor of high-quality black tea exhibits a reddish hue, a lingering aromatic profile, and a sweet, mellow taste. The contents of various odor components in black teas from different origins are considerably different [13,14]; however, the effects of these differences on the presentation of distinctive odor characteristics in various products have not yet been fully elucidated.

Therefore, we aimed to reveal the relationship between odor components and the odor characteristics of DH, KH, and CH; to this end, we conducted a sensory evaluation and electronic nose analysis to analyze their odor characteristics, and a solid-phase microextraction method was used to extract volatile compounds from the three teas for analyses. Statistical methods were used to screen marker compounds that can characterize the odor characteristics of the three teas and determine their flavor differences [15,16]. The results of this study provide a reference for consumers to choose black tea and contribute to a comprehensive understanding of the odor quality of black tea products. This study provides an in-depth analysis of the flavor characteristics and compound composition of several black teas, offering a reference for consumers to choose black tea, contributing to a comprehensive understanding of the odor quality of black tea products, and providing a theoretical basis for the research and development of more black tea products.

## 2. Results and Discussion

### 2.1. Quantitative Descriptive Analysis (QDA)

**Figure 1:**
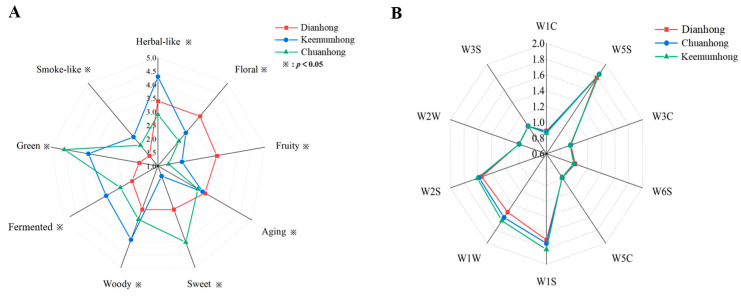
Aroma profile of Keemumhong, Chuanhong, and Dianhong (**A**). Electronic nose radar diagram of Dianhong, Chuanhong, and Keemumhong (W1C, mainly sensitive to aromatic components and benzene compounds; W5S, mainly sensitive to nitrous oxides; W3C, mainly sensitive to ammonia and aromatic components; W6S, mainly sensitive to hydrocarbons; W5C mainly sensitive to alkane, aromatics, and small polar compounds; W1S, broad range—methane; W1W, mainly sensitive to inorganic sulfides; W2S, mainly sensitive to most alcohols, aldehydes, and ketones; W2W, mainly sensitive to aromatics and organic sulfides; W3S, mainly sensitive to long-chain alkanes) (**B**).

A sensory evaluation of the three black teas was performed. Referring to the China Tea Industry Standard T/CTSS 58-2022 [17] Tea Sensory Flavor Wheel, the main description words were selected to quantitatively describe the odor attributes of CH, DH, and KH and are depicted using a radar diagram (Figure 1A). Each sample showed strong herbal-like, aging, and woody odors. DH was mainly characterized by floral, fruity, herbal-like, and aging odors; CH was mainly characterized by sweet and green odors; and KH was mainly characterized by herbal-like, woody, green, and fermented odors. From the QDA results, the odor intensities of sweet, woody, floral, fruity, and green were significantly different among the different black teas. DH scored the highest for floral and fruity odors, KH scored the highest for woody, fermented, and herbal-like odors, and CH scored the highest for sweet and green odors. There were significant differences in the odor characteristics of the three samples.

### 2.2. E-Nose Analysis Results

Analysis of the three black teas using an E-nose (Figure 1B) showed that among the 10 transducers, the W1S, W1W, W2S, and W5S transducers showed high responses to the odor compounds, indicating that the three black tea samples contained high concentrations of methyl compounds, sulfur-containing compounds, alcohols, aldehydes, and ketones, and nitrogen oxides. The lowest response value was W1C, which indicates that the aromatic compound content was lower. However, the transducers’ response intensity contours of the three black teas were similar, with some differences only in the response intensities of three transducers W1W, W1S, and W2S; the contents of methyl compounds, sulfur compounds, alcohols, aldehydes, and ketones were higher in KH than in CH and DH. This result also suggests that the differences in the odor characteristics of the three black teas cannot be effectively distinguished through sensory evaluation by E-nose analysis alone. A detailed analysis of the volatile odor components in the samples is required to explain the different odor characteristics of the three black teas at a molecular level.

### 2.3. Analysis of Volatile Compounds in Black Tea of Different Origins

**Figure 2:**
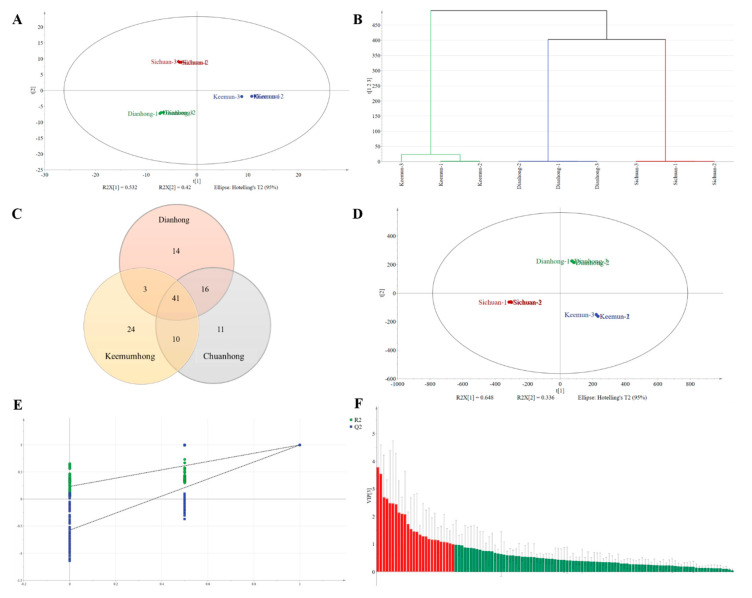
PCA of volatile compounds in Dianhong, Chuanhong, and Keemumhong (**A**). HCA of volatile compounds in Dianhong, Chuanhong, and Keemumhong (**B**). Venn diagram of volatile compounds in Dianhong, Chuanhong, and Keemumhong (**C**). PLS-DA scores for volatile compounds of Dianhong, Chuanhong, and Keemumhong (**D**). PLS-DA permutation test for volatile compounds of Dianhong, Chuanhong, and Keemumhong (**E**). PLS-DA VIP scores for volatile compounds of Dianhong, Chuanhong, and Keemumhong (**F**).

**Figure 3:**
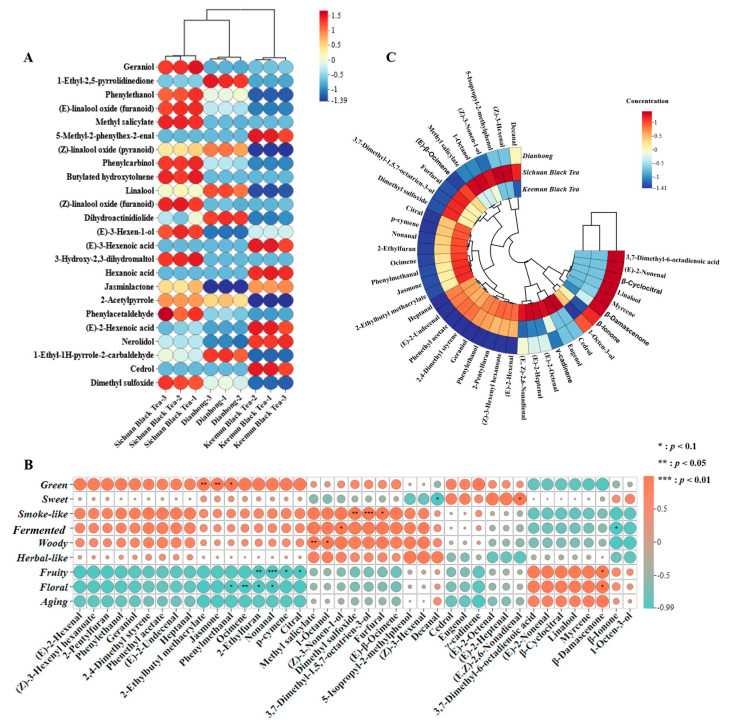
The heat map of key identifying compounds in Dianhong, Chuanhong, and Keemumhong (**A**). Pearson’s correlation analysis of 41 odor compounds and flavor profiles with OAV > 1 (**B**). Comparison of quantitative compound contents in Dianhong, Chuanhong, and Keemumhong (**C**).

Overall Analysis of Volatile Compound Composition in Black Tea. The volatile compounds in the samples were extracted using the solid-phase microextraction (SPME) method and then analyzed qualitatively using GC×GC-MS and quantitatively using GC-MS; in total, 119 volatile compounds were identified in the three black tea samples, including 19 aldehydes, 23 alcohols, 10 esters, 2 acids, 16 alkanes, 17 ketones, 5 phenols, 15 heterocyclic compounds, and 12 other compounds. Alcohols, aldehydes, and esters are the main volatile compounds present in black tea. The volatile contents of the different species showed considerable differences. Multivariate statistical analysis of the 119 volatile compounds was performed to understand the differences and similarities between different black teas. First, black tea was analyzed using both principal component analysis (PCA) and hierarchical clustering analysis (HCA) based on the content of the identified volatile compounds. Figure 2A shows the PCA results (PC1 = 53.2%; PC2 = 42.0%), which indicates that DH and CH were closely distributed. The three samples were significantly divided along PC1. The clustering results of HCA (Figure 2B) were consistent with the trends shown by PCA. The Venn diagram (Figure 2C) shows that DH contains 14 unique compounds, CH—11 unique compounds, and KH—24 unique compounds; additionally, 41 volatile compounds were common in the three black teas. The underlying causes of these similarities and differences can be attributed to various factors, including raw materials, geographical factors, and processing techniques [18,19,20]. The partial least squares discriminant analysis (PLS-DA) model (Figure 2D) effectively divided the three different black teas with good predictive ability (R2Y = 0.648, Q2 = 0.336) and no signs of overfitting (Figure 2E). According to variable important in projection (VIP) > 1.0 and *p* < 0.05, 24 compounds were identified as the key discriminating compounds of the three black teas (Figure 3A), and the 24 compounds were subjected to heat map analysis (Figure 3B). Compared with the other two black teas, CH mainly comprised alcohols, aldehydes, and esters, which is consistent with the main odor compounds of black teas in previous reports [21,22]. Linalool and its oxides were the key identifying compounds of DH, while the key identifying compounds of KH included aldehydes, acids, alcohols, and esters.

### 2.4. Screening of Three Black Teas’ Key Odor Compounds

The contribution of volatiles to the odor of black tea is not only closely related to their concentration but also depends on their odor thresholds [23]. OAV is used to measure the odor intensity of each compound, and compounds with an OAV > 1 are usually considered important odor-active components [24], with a larger corresponding OAV value representing a greater compound odor intensity [25]. After quantitative analysis by GC-MS, we identified 41 volatile compounds with an OAV of >1 (Table 1). Among them, eight compounds, linalool (floral), geraniol (rose-like and citrus-like), (*E*)-2-octenal (fatty and nutty), *β*-damascenone (fruity, floral, and sweet), *β*-ionone (floral), methyl salicylate (mint-like), *γ*-cadinene (woody), and dimethyl sulfoxide (fatty) had OAV values >50. The odor compounds with high OAV values mainly exhibited floral, fruity, sweet, and woody attributes, which was consistent with the trend of the sensory evaluation, indicating that they make an important contribution to the basic odor characteristics of black tea. Furthermore, we discussed the relationship between the odor characteristics of the samples and the compounds. Pearson’s correlation analysis (Figure 3B) was performed on 41 odor compounds and the flavor profiles with an OAV > 1. The results showed that the green correlation compounds were 2-ethylbutyl methacrylate (*p* < 0.01), jasmone (*p* < 0.01), and phenylethanol (*p* < 0.05), with esters and alcohols being highly correlated; the sweet-related compounds were decanal (*p* < 0.05) and (*E*, *Z*)-2,6-nonadienal (*p* < 0.05), with the highly correlated compounds being aldehydes; the smoke-like compounds were dimethyl sulfoxide (*p* < 0.01), 3,7-dimethyl-1,5,7-octatrien-3-ol (*p* < 0.001), and furfural (*p* < 0.05), with the highly correlated compounds being alcohols and aldehydes; the fermentation-related odor compounds were (*Z*)-3-nonen-1-ol (*p* < 0.05) and *β*-ionone (*p* < 0.05), with the highly relevant odor compounds being alcohols and ketones; the woody odor compounds were methyl salicylate (*p* < 0.01) and 1-octanol (*p* < 0.05), with the highly relevant compounds being esters and alcohols; the floral odor compounds were phenylethanol (*p* < 0.05), ocimene (*p* < 0.01), nonanal (*p* < 0.05), 2-ethylfuran (*p* < 0.05), and *β*-damascenone (*p* < 0.05), with highly relative compounds being alkenes, alcohols, aldehydes, and ketones; and the fruity odor compounds were 2-ethylfuran (*p* < 0.01), nonanal (*p* < 0.001), *p*-cymene (*p* < 0.05), citral (*p* < 0.05), and *β*-damascenone (*p* < 0.05), with highly relevant compounds being aldehydes. Relevant compounds were not detected in herbal-like, smoke-like, or aging odors, and follow-up testing was performed using other detection methods.

#### 2.4.1. Key Odor Compounds of DH

Comparing the quantitative compound contents of each black tea, the results show (see Figure 3C) that for DH, the whole compound contents were lower than those of KH and CH. DH had eight key odor compounds whose contents were higher than those in the other black teas, including 1-octen-3-ol, linalool, (*E*)-2-nonenal, *β*-cyclocitral, *β*-damascenone, *β*-ionone, 3,7-dimethyl-6-octadienoic acid, and myrcene, which mainly contribute to the floral, fruity, fresh, and woody odors. Linalool, geraniol, *β*-damascenone, *β*-ionone, and methyl salicylate, with OAV values > 50, are important key odor compounds in DH. Among these compounds, alcohols have floral and sweet flavors. The linalool content of DH was significantly higher than that of the other black tea samples. Linalool, a common compound in black tea, imparts a floral odor to black tea.

#### 2.4.2. Key Odor Compounds of KH

Among the 41 odor compounds identified in black tea, 44% of the compounds in KH demonstrated higher contents than those in both DH and CH. Geraniol, *β*-damascenone, methyl salicylate, and dimethyl sulfoxide all had OAV values of >50, which mainly led to floral, fruity, fresh, and fatty odors. KH is a typical representative of floral-flavored black tea, and many of its key odorous compounds are floral. Geraniol is one of the most common alcohol compounds in black tea and is regarded as the key odorant in four of the most famous black teas in the world [22,27]. Together with linalool and methyl salicylate, geraniol provides outstanding floral benefits for KH [28]. In addition, the above analysis showed that KH had the highest geraniol content and OAV value.

#### 2.4.3. Key Odor Compounds of CH

The contents of the 14 key odor compounds in CH were higher than those in DH and KH. Four compounds, including geraniol, *β*-damascenone, methyl salicylate, and dimethyl sulfoxide, had OAV values of >50. CH had the same odor as KH: floral, fruity, fresh, and fatty. Except for the OAV value of geraniol, which was slightly higher than that of KH, the OAV values of the other compounds were lower than those of KH.

### 2.5. Screening of Key Odor Compounds of Black Tea

In general (see Figure 3C), linalool, geraniol, (*E*)-2-octenal, *β*-damascenone, *β*-ionone, methyl salicylate, and dimethyl sulfoxide had the most prominent OAV values, of which linalool, geraniol, *β*-damascenone, and methyl salicylate were the most important key odor compounds that provide rich floral, fruity, and fresh odors to black tea. These odors constitute the typical characteristics of black tea, consistent with the results of previous reports [28].

## 3. Materials and Methods

### 3.1. Black Tea Samples and Breakage Processing

The three black teas used in this study were of superfine grade. DH was purchased from Yunnan Dianhong Group Co., Ltd., located in Lincang City, Yunnan Province, China. CH was purchased from Yibin Chuanhong Group Co., Ltd., located in Yibin City, Sichuan Province, China. KH was purchased from Keemun Qiya Tea Responsibility Company, located in Huangshan City, Anhui Province, China. Black tea was pulverized with liquid nitrogen using a pulverizer (10 s, three times each) to avoid compositional changes due to frictional warming during milling. The crushed black teas were stored in a −80 °C refrigerator before analysis.

### 3.2. Chemicals

The following chemicals (>99%) were used in this study: dichloromethane, hexane, anhydrous sodium sulfate, 1-octen-3-ol, linalool, 1-octanol, 3,7-dimethyl-1,5,7-octatrien-3-ol, (*Z*)-3-hexenyl hexanoate, (*Z*)-3-nonen-1-ol, geraniol, phenylethanol, (Z)-3-hexenal, heptanal, (*E*)-2-hexenal, (*E*)-2-heptenal, nonanal, (*E*)-2-octenal, furfural, (*E*)-2-nonenal, decanal, phenylmethanal, (*E*,*Z*)-2,6-nonadienal, *β*-cyclocitral, citral, (*E*)-2-undecenal, *β*-damascenone, *β*-ionone, jasmone, methyl salicylate, phenethyl acetate, 3,7-dimethyl-6-octadienoic acid, eugenol, 5-isopropyl-2-methylphenol, 2-ethylfuran, 2-pentylfuran, myrcene, 2-ethylbutyl methacrylate, ocimene, (*E*)-*β*-ocimene, *p*-cymene, 2,4-pimethyl styrene, *γ*-cadinene, dimethyl sulfoxide, and cedrol, which were purchased from Pinellia Technology Co., Ltd. (Beijing, China). Chromatography-grade pure chemicals, including 1,2-dichlorobenzene and n-alkanes (C7-C30), were purchased from Sigma-Aldrich Inc. (St. Louis, MO, USA). High-purity helium (99.999% purity) and nitrogen (99.99% purity) were purchased from Beijing Tianli Renhe Trading Co., Ltd. (Beijing, China).

### 3.3. Sensory Evaluation

The sensory evaluation was carried out based on the China Tea Industry Standard T/CTSS 58-2022. The evaluator group consisted of 20 panelists aged 22–26 years, with a male-to-female ratio of 1:1. Each member received training before formal tasting of the samples, including basic knowledge of sensory analysis, identification of odor characteristics, accurate evaluation processes, and the establishment and application of scales. Quantitative description analysis (QDA) was used to obtain a complete description of the sensory attributes of the black tea samples using a 10-point intensity scale (0 = none; 1–3 = weak; 4–6 = moderate; 7–9 = strong). The panelists scored each sample based on the intensity of nine odor attributes, including floral, fruity, sweet, woody, green, herbal-like, fermented, smoke-like, and aging. The average of the scores of all the panelists was considered the final result, and the odor profiles of odor characteristics were drawn. All the panelists fully understood the sensory evaluation plan and signed an authorization letter for the use of their personal data. The scientific ethics review of this study was approved by the Life Science Ethics Committee of Yunnan Agricultural University (approval No. 202312002).

### 3.4. E-Nose Analysis

E-nose analysis was performed using a PEN 3.5 E-nose device (Winmuster Airsense Analytics Inc., Schwerin, Germany) containing 10 metal-oxide semiconductors with different chemical compositions and thicknesses for the detection of the corresponding compounds. First, 1.0 g of each black tea sample was weighed and placed in a 40 mL headspace bottle, 10 mL of ultrapure water was added, and the samples were held in a water bath at 45 °C. The parameters were set as follows: chamber flow rate of 300 mL/min; sample purge flow rate of 300 mL/min; rinsing time of 100 s; and detection time of 180 s. The cycles were repeated eight times for each sample. Data processing was performed using Winmuster E-nose software, version 1.6.2.

### 3.5. Extraction of Odor Compounds

We placed 1 g of black tea powder sample into a 40 mL headspace bottle, 10 mL of distilled water was added, and the sample was equilibrated in a water bath at 55 °C for 10 min. Extraction was performed for 50 min, with the inlet set at 230 °C, and resolution 5 min after the insertion of the extraction head. The samples were then analyzed by GC×GC-MS.

### 3.6. GC×GC-MS Analysis

An Agilent GC-MS instrument (8890-5977B, Santa Clara, CA, USA) was used for the GC×GC-MS analysis. The first polar column DB-Wax (30 m × 0.25 mm, 0.25 μm film thickness; J&W Scientific, Santa Clara, CA, USA) and the second mid-polar column DB-17 ms (1.85 m × 0.18 mm, 0.18 μm film thickness; J&W Scientific) were set inside the same GC oven. Ultrahigh-purity helium (99.999%) was used as the carrier gas at a constant flow rate of 1.5 mL/min. Electron ionization was used with an electron energy of 70 eV at 200 °C and an m/z scanning range of 40–350. Injections were carried out in splitless mode at 230 °C. The column program was maintained at 40 °C for the first 5 min, then increased to 200 °C at a rate of 4 °C/min, and held at this temperature for 8 min. Compounds were identified by comparing their mass spectra and GC linear retention index (RI) with those of the reference compounds using GC×GC-MS. N-alkanes (C7–C25) were used to determine the RI of each compound on the DB-Wax column.

### 3.7. Quantitative Analysis and Calculation of the Odor Activity Value (OAV)

The volatile compounds in the samples were quantified using GC-MS in SIM mode, and 1 μL of 2-methyl-3-heptanone (0.816 μg/μL) was added as an internal standard. Relative quantification of the finalized odor compounds was carried out using the internal standard method, and the relative contents of the corresponding compounds were calculated from the ratio of volatile components and the peak area of the internal standard.

The OAV represents the odor intensity of a compound. The OAV was calculated by dividing the relative concentration of each compound by its odor threshold in water.

### 3.8. Statistical Analyses

Each experiment was repeated three times, and the associated results are expressed as the mean ± standard deviation (SD). All tables were created using Microsoft Office Excel 2019, and SPSS 25.0 software was used for the one-way analysis of variance (ANOVA), Duncan’s multiple range test (*p* < 0.05) to determine significant differences, and Pearson’s correlation analysis. SIMCA 14.1 was used for the PCA, HCA, and PLS-DA.

## 4. Conclusions

The results of the sensory evaluation showed that DH was mainly characterized by sweet, floral, fruity, and fermentation odors, whereas CH and KH were mainly characterized by sweet and herbal-like odors. The volatile metabolites of different varieties of black tea and the correlation between volatile metabolites and sensory attributes were analyzed; 119 volatile compounds were identified by GC×GC-MS analysis. Twenty-four key compounds in DH, KH, and CH samples were screened using multivariate statistical analysis. The content of volatile compounds was obtained by quantitative analysis using GC-MS; 41 key odor compounds with OAV values >1 were screened by the OAV value calculation, and the relationships with sensory attributes were analyzed by Pearson’s correlation analysis. Among the 41 key odor-active compounds, 8 compounds had OAV values >50, including linalool (floral, OAV = 26–54), geraniol (rose-like and citrus-like, OAV = 274–1146), 2-octenal (fatty and nutty, OAV = 45–138), *β*-damascenone (fruity, floral, and sweet, OAV = 356–1431), *β*-ionone (floral, 6737–3864), methyl salicylate (mint-like, OAV = 119–569), *γ*-cadinene (woody, OAV = 25–67), and dimethyl sulfoxide (fatty, OAV = 70–122), which mostly exhibit floral, fruity, fresh, and woody odors, leading to the main odor of black tea. The findings of this study contribute to a comprehensive understanding of the odor quality of black tea products and the establishment of a black tea quality evaluation system based on odor characteristics, and provide a reference for consumers to choose black tea.

## Figures and Tables

**Table 1:** The OAV values of key volatile compounds in Dianhong, Keemunhong, Chuanhong.

Compounds	Odor Threshold ^1^/(µg/L)	Odor	Concentration ^2^ (±SD ^3^) (μg/kg)/OAV ^4^		
			Dianhong	Chuanhong	Keemunhong
1-Octen-3-ol	6	mushroom-like	98.7 ± 18.0 ^a^ (16.45)	79.4 ± 5.0 ^a^ (13.23)	94.3 ± 5.6 ^a^ (15.71)
Linalool	150	floral	8081.5 ± 141.5 ^a^ (53.88)	3921.4 ± 361.5 ^b^ (26.14)	3842.0 ± 141.1 ^b^ (25.61)
1-Octanol	110	floral	73.3 ± 4.4 ^c^ (<1)	161.7 ± 4.3 ^a^ (1.47)	100.0 ± 6.5 ^b^ (<1)
3,7-Dimethyl-1,5,7-octatrien-3-ol	110	green	272.9 ± 26.4 ^c^ (2.48)	702.5 ± 48.5 ^a^ (6.39)	517.5 ± 18.0 ^b^ (4.7)
(*Z*)-3-Hexenyl hexanoate	12.1	fruity	—	238.7 ± 28.2 ^b^ (19.73)	282.2 ± 10.7 ^a^ (23.32)
(*Z*)-3-Nonen-1-ol	1	fresh, green	10.3 ± 0.5 ^b^ (10.27)	33.4 ± 2.9 ^a^ (33.39)	20.2 ± 0.4 ^b^ (20.23)
Geraniol	7.5	rose-like, citrus-like	2056.0 ± 79.1 ^c^ (274.14)	8306.5 ± 232.8 ^b^ (1107.53)	8595.2 ± 355.2 ^a^ (1146.03)
Phenylethanol	1000	floral	784.0 ± 56.9 ^b^ (<1)	1158.8 ± 45.6 ^a^ (1.16)	1187.4 ± 83.4 ^a^ (1.19)
(*Z*)-3-Hexenal	0.25	fruity	—	3.6 ± 0.2 (14.43)	—
Heptanal	6.1	citrus-like, fatty	—	9.1 ± 1.2 ^a^ (1.49)	8.5 ± 1.6 ^a^ (1.4)
(*E*)-2-Hexenal	110	grassy, fatty	40.7 ± 1.6 ^c^ (<1)	127.7 ± 4.4 ^b^ (1.16)	142.7 ± 5.8 ^a^ (1.3)
(*E*)-2-Heptenal	13	fatty, green	14.4 ± 1.4 ^b^ (1.11)	—	43.5 ± 13.1 ^a^ (3.34)
Nonanal	260	citrus-like, green	162.8 ± 20.1 ^b^ (<1)	262.8 ± 18.5 ^a^ (1.01)	299.3 ± 18.1 ^a^ (1.15)
(*E*)-2-Octenal	1.7	fatty, nutty	77.0 ± 3.5 ^b^ (45.32)	—	234.0 ± 13.9 ^a^ (137.67)
Furfural	282	nutty, sweet	549.9 ± 5.0 ^c^ (1.95)	1251.5 ± 52.3 ^a^ (4.44)	938.2 ± 45.5 ^b^ (3.33)
(*E*)-2-Nonenal	0.15	fatty, green	7.5 ± 0.6 (49.74)	—	—
Decanal	6.2	fatty, fruity	11.5 ± 2.8 ^b^ (1.86)	21.4 ± 1.5 ^a^ (3.45)	—
Phenylmethanal	3500	almond-like	3892.3 ± 178.6 ^c^ (1.11)	4561.5 ± 520.8 ^b^ (1.3)	4994.3 ± 671.5 ^a^ (1.43)
(*E*,*Z*)-2,6-Nonadienal	5	green, fatty	4.3 ± 0.2 ^b^ (<1)	—	9.4 ± 0.4 ^a^ (1.89)
*β*-Cyclocitral	32	lemon-like	179.2 ± 23.5 ^a^ (5.6)	134.4 ± 5.7 ^b^ (4.2)	134.2 ± 3.3 ^b^ (4.19)
Citral	85	fruity, lemon-like	120.6 ± 20.6 ^b^ (1.42)	408.4 ± 7.5 ^a^ (4.81)	490.9 ± 116.6 ^a^ (5.77)
(*E*)-2-Undecenal	1.4	fruity	—	26.1 ± 4.9 ^a^ (18.63)	24.0 ± 1.1 ^a^ (17.12)
*β*-Damascenone	0.05	fruity, floral, sweet	71.5 ± 9.1 ^a^ (1430.85)	33.9 ± 3.6 ^b^ (677.77)	18.3 ± 1.5 ^c^ (365.23)
*β*-Ionone	0.1	floral	673.7 ± 52.2 ^a^ (6736.68)	—	386.4 ± 5.7 ^b^ (3864.06)
Jasmone	7	floral, tea-like	44.3 ± 4.8 ^c^ (6.33)	96.9 ± 11.0 ^b^ (13.84)	133.8 ± 13.5 ^a^ (19.12)
Methyl salicylate	40	mint-like	4751.7 ± 711.4 ^c^ (118.79)	22,770.5 ± 1010.8 ^a^ (569.26)	10,628.7 ± 499.5 ^b^ (265.72)
Phenethyl acetate	19	floral	10.4 ± 1.3 ^b^ (<1)	19.5 ± 0.9 ^a^ (1.03)	18.3 ± 1.3 ^a^ (<1)
3,7-Dimethyl-6-octadienoic acid	23	green, woody	101.3 ± 16.6 (4.4)	—	—
Eugenol	1.8	clove-like, woody	—	—	5.7 ± 0.8(3.14)
5-Isopropyl-2-methylphenol	1.8	woody	—	5.7 ± 1.1 (3.19)	—
2-Ethylfuran	17	earthy, malty	7.5 ± 2.5 ^c^ (<1)	40.3 ± 3.5 ^b^ (2.37)	53.2 ± 1.7 ^a^ (3.13)
2-Pentylfuran	6	fruity, green	12.9 ± 1.0 ^a^ (<1)	14.9 ± 4.7 ^a^ (2.48)	15.3 ± 3.0 ^a^ (2.55)
Myrcene	15	woody, fruity	268.6 ± 7.5 ^a^ (17.9)	112.8 ± 27.1 ^b^ (7.52)	99.5 ± 44.2 ^b^ (6.63)
2-Ethylbutyl methacrylate	34	lemon-like	32.7 ± 0.8 ^c^ (<1)	48.8 ± 3.5 ^b^ (1.44)	60.0 ± 4.4 ^a^ (1.76)
Ocimene	34	citrus-like, green	3.4 ± 0.7 ^c^ (<1)	352.8 ± 31.4 ^b^ (10.38)	534.8 ± 27.0 ^a^ (15.73)
(*E*)-*β*-Ocimene	60	sweet, herbal-like	24.6 ± 3.4 ^b^ (<1)	218.1 ± 34.2 ^a^ (3.64)	157.3 ± 56.7 ^a^ (2.62)
*p*-Cymene	100	citrus-like, woody	63.1 ± 9.0 ^b^ (<1)	118.4 ± 6.6 ^a^ (1.18)	134.1 ± 10.5 ^a^ (1.34)
2,4-Dimethyl styrene	85	spicy, balsamic	63.3 ± 10.1 ^b^ (<1)	105.1 ± 4.3 ^a^ (1.24)	100.5 ± 5.5 ^a^ (1.18)
*γ*-Cadinene	1.5	woody	—	38.0 ± 1.2 ^b^ (25.34)	100.1 ± 5.2 ^a^ (66.71)
Dimethyl sulfoxide	0.3	fatty	—	36.5 ± 5.2 ^a^ (121.62)	21.0 ± 2.6 ^b^ (70.13)
Cedrol	0.5	woody	—	—	20.9 ± 2.8 (41.8)

^1^ Thresholds of compounds are found in the *Odor Thresholds Compilations of Odor Threshold Values in Air, Water, and Other Media (Second Enlarged and Revised Edition)*, where the thresholds are carried out according to the physical and chemical indicators of black tea, and the thresholds of compounds are determined [26]. ^2^ Different letters in the same row indicate significant differences (*p* < 0.05) between the concentrations of the compounds in Dianhong, Keemunhong, and Chuanhong. ^3^ SD, standard deviation. ^4^ Odor active value, calculated by dividing the concentrations by the odor thresholds.

## Data Availability

Data are contained within the article.

## Abbreviations

The following abbreviations are used in this manuscript:

DH	Dianhong
CH	Chuanhong
KH	Keemunhong
GC×GC-MS	Comprehensive two-dimensional gas chromatography–mass spectrometry
OAV	Odor activity value
QDA	Quantitative descriptive analysis
SPME	Solid-phase microextraction
GC-MS	Gas chromatography–mass spectrometry
PLS-DA	Partial least squares discriminant analysis
HCA	Hierarchical clustering analysis
PCA	Principal component analysis
VIP	Variable important in projection
SD	Standard deviation
ANOVA	Analysis of variance
